# Association between body esteem and sugar-sweetened beverage intake among Chinese undergraduate students: a cross-sectional study

**DOI:** 10.3389/fnut.2024.1465518

**Published:** 2025-01-14

**Authors:** Jiawen Zhu, Yuanyuan Zhu, Zihe Zhao, Qianling Huang, Changju Liu, Zhi Zeng

**Affiliations:** ^1^School of Health Economics and Management, Nanjing University of Chinese Medicine, Nanjing, China; ^2^School of Nursing, Nanjing University of Chinese Medicine, Nanjing, China; ^3^Department of Endocrinology, Affiliated Jiangning Hospital of Nanjing Medical University, Nanjing, China

**Keywords:** body esteem, sugar-sweetened beverage, undergraduate students, China, cross-sectional study

## Abstract

**Background:**

High intake of sugar-sweetened beverages has been linked to a range of physical, psychological, and emotional issues. Although there were various factors influencing sugar-sweetened beverage intake, the relationship between body esteem and sugar-sweetened beverage intake remains unclear. This study aimed to investigate the association between three dimensions of body esteem (body esteem-appearance, body esteem-attribution, and body esteem-weight) and the likelihood of high sugar-sweetened beverage intake.

**Methods:**

A cross-sectional study was conducted among undergraduate students at Nanjing University of Chinese Medicine. Logistic regression analyses were used to assess the association between the three dimensions of body esteem and the risk of high sugar-sweetened beverage intake. Additionally, restricted cubic splines and subgroup analyses were implemented to further explore the associations.

**Results:**

A total of 969 participants were included in the study, with 771 females (79.6%). The mean age of the participants was 20.07 years (standard deviation [SD] = 1.65). After adjusting for covariates, body esteem-appearance was found to be negatively associated with high sugar-sweetened beverage intake (OR = 0.962, 95% CI = 0.935–0.989, *p* = 0.007), while body esteem-attribution was positively associated with high sugar-sweetened beverage intake (OR = 1.091, 95% CI = 1.046–1.139, *p* < 0.001). However, no significant association was found between body esteem-weight and high sugar-sweetened beverage intake (*p* = 0.781). Restricted cubic spline plots showed no non-linear associations between any dimensions of body esteem and the risk of high sugar-sweetened beverage intake (*p*-nonlinear was 0.912, 0.225, 0.109, respectively). Subgroup analyses revealed no significant interactions.

**Conclusion:**

These findings underscored the significance of targeted health promotion strategies and provided references for educational institutions or governmental bodies to steer undergraduate beverage consumption toward healthier patterns.

## 1 Introduction

World Health Organization defines a beverage containing free sugars as a sugar-sweetened beverage, recommends limiting free sugar intake to less than 10% of total energy intake, preferably less than 5% (1). According to the dietary guidelines for Chinese residents (2022), sugar-sweetened beverages are beverages with more than 5% artificially added sugar (≥5 g/100 g) during production, such as soft drinks, fruit juices, sports drinks, sugar-sweetened coffee, and milk tea (2). Recent trends indicate a decline in sugar-sweetened beverage consumption in high-income countries but a rise in low-and middle-income countries (3). In China, students gain more autonomy over their diet after entering university (4). Fifty-two point 7 % of Chinese college students report high emotional eating (5). The diet quality of Chinese college students is relatively poor, characterized by higher consumption of fried foods and high-fat foods, and lower consumption of fish, fruits, and vegetables (6). Sugar-sweetened beverages are also becoming increasingly popular among Chinese college students; 12.2% of them consume sugar-sweetened beverages ≥ 2 times a week (7). Carbonated beverages, fruit beverages, tea beverages, energy beverages, milk beverages, and milk tea are the most favored among Chinese college students (8). Positive perceptions of sugar-sweetened beverages include their delicious taste, invigorating nature, and associations with pleasant events, while negative perceptions involve their potential in the development of various diseases (9). High sugar-sweetened beverage intake can lead to physical disorders such as hypertension, type 2 diabetes mellitus, and obesity (10), alongside psychological and emotional problems such as depression (11), and distress (12).

In the study on the impact of attitudes toward beverages, “self-image” are identified as major contributors to the excessive sugar-sweetened beverage intake and low water intake among Mexican adults (13). Meanwhile, labeling sugar-sweetened beverage with warnings about negative perceptions of bodies can reduce public purchases (14). Female adults with high body appreciation and low body shame typically exhibit low dietary restriction (15). Sugar-sweetened beverage intake, as a dietary behavior, may be related to body esteem.

Body esteem refers to individuals’ satisfaction with various aspects of their bodies, reflecting feelings about their bodies and influenced by self-worth and social evaluation (16). This study focuses on three aspects of body esteem, namely overall perceptions of appearance, social evaluations attributed to one’s body and appearance, and weight satisfaction, which are defined as body esteem-appearance, body esteem-attribution and body esteem-weight, respectively (16). Body esteem constitutes a crucial element of global self-esteem, and individuals with lower global self-esteem are usually accompanied by diminished body esteem (17). Individuals with high levels of body esteem often exhibit healthier eating attitudes (18), improved diet quality, greater intuitive eating tendencies, and reduced emotional eating (19). However, individuals with low levels of body esteem, characterized by appearance dissatisfaction and weight dissatisfaction, are more likely to overeat, increasing the risk of eating disorder (20). In addition, body esteem is associated with other health behaviors, including physical activity (17) and e-cigarette use (21).

Self-determination theory investigates the influence of psychological needs on health behaviors (22). Previous studies have already employed self-determination theory to elucidate dietary behaviors. The positive associations between self-efficacy, self-compassion, perceived support and healthy eating have been substantiated in adult populations with type 2 diabetes and female undergraduate students (23–25). The psychological variables involved in previous studies only focus on the three basic psychological needs: autonomy (feeling that self is the source of one’s actions), competence (feeling mastery or effectiveness), and relatedness (feeling connected to others in a meaningful way). Body esteem is somewhat linked to relatedness, as individuals’ body satisfaction is influenced by external evaluations and involves some connection with others. However, body esteem is a complex concept that plays a significant role in both self-identity and social interactions. Body esteem, as a psychological need, does not belong to any of the three basic psychological needs and wasn’t fully taken into consideration within the self-determination theory. Combined with the prior academic role of body esteem, we found that body esteem can influence dietary behaviors (19, 20). We introduced body esteem into self-determination theory framework, expanding self-determination theory’s dimensions of psychological needs. Theoretically, this expansion facilitates the revelation of complex impact of various psychological factors on health behaviors. Practically, recognizing the potential role of body esteem can provide a foundation for developing health intervention strategies, aiding individuals in improving their dietary habits. Self-determination theory suggests that fulfilling individuals’ positive psychological needs can promote healthy behaviors (22). Body esteem can be regarded as a positive psychological need, while high sugar-sweetened beverage intake can be seen as an unhealthy eating behavior. Hence, within the framework of self-determination theory, we hypothesized that body esteem is negatively associated with the risk of high sugar-sweetened beverage intake among Chinese undergraduate students.

Understanding the association between body esteem and sugar-sweetened beverage intake is particularly significant for the undergraduate students. The undergraduate years represent a critical period for young individuals transitioning into adulthood, wherein they become increasingly sensitive to their bodies. Chinese college students typically achieve satisfactory body images through dress, appearance, and balanced diets, in contrast to the emphasis on physical exercise among Western counterparts (26). Surprisingly, Chinese college students are increasingly managing their bodies based on external evaluations (26). Sugar-sweetened beverages are one of the main sources of energy intake for college students (27). However, excessive sugar-sweetened beverage intake can not only lead to various mental health challenges, including emotional, behavioral, and social adjustment difficulties (7), but also detrimentally impact sleep quality and daytime cognitive performance (28). Considering these adverse effects, it is crucial to control sugar-sweetened beverage intake. A study among medical students found that all dimensions of male body esteem, including physical attractiveness, upper body strength, and physical condition, are positively associated with diet quality. For females, only the physical condition dimension of body esteem is positively associated with diet quality (29). Previous study has demonstrated the importance of psychological interventions in improving dietary quality (30). Existing studies have typically examined the relationship between body esteem and sugar-sweetened beverages from a broad or limited perspective. Moreover, most studies focus on the general population, often overlooking specific groups like undergraduate students. Given the differences in cultural backgrounds and eating habits, cross-cultural studies are also needed. Therefore, based on the framework of self-determination theory, this study aimed to explore the relationship between the three dimensions of body esteem (body esteem-appearance, body esteem-attribution, and body esteem-weight) and sugar-sweetened beverage intake among Chinese undergraduate students. It addresses gaps related to cultural and demographic differences, offering valuable insights for future health interventions.

## 2 Materials and methods

### 2.1 Data collection and participants

Data for this study were derived from a cross-sectional survey project conducted between September 2021 and May 2022 among undergraduate students enrolled at Nanjing University of Chinese Medicine. This project primarily examines the relationship between psychological health and dietary habits, focusing on body image, weight self-perception, beverage consumption, and intuitive eating. Utilizing a convenience sampling approach, we recruited participants in several ways. After explaining the research purpose to the counselors, they assisted in recruiting participants. Additionally, researchers promoted the study by putting up posters on campus and advertising on social media. Prior to the survey, researchers were professionally trained. The questionnaire was improved after conducting a preliminary survey with a class of students. Researchers gathered the recruited participants in classrooms and explained the research purpose again, focusing on highlighting items that were prone to misunderstanding. Participants used their phones to scan the QR code for the questionnaire and completed it independently. The electronic questionnaires were collected through the “Wenjuanxing” platform.^1^ Prior to participation, all individuals provided informed consent. We ensured that all data was collected anonymously. The sample size was determined using G*Power 3.1.9.7 software with the following assumptions: a two-sided significance level of 0.05, a Power of 80%, and an Odds ratio of 0.8 (31). The estimated sample size for this study was 853 individuals. Inclusion criteria were as follows: (1) undergraduate students currently enrolled at the university, in (2) those with good physical condition, and (3) those willing to participate voluntarily. A total of 1,159 undergraduate students participated initially. Exclusion criteria included (1) individuals with communication disorders, cognitive dysfunction, or eating disorder with a SCOFF questionnaire score of 2 or more (32), (2) those on course suspension, (3) those experiencing weight loss due to physical illnesses, and (4) those failing to complete the questionnaire (e.g., omission, duplication, carelessness, or ghostwriting). Participants meeting any exclusion criteria were excluded from the study. Following quality control measures, 190 questionnaires were deemed incomplete, careless, or risk of eating disorder and thus excluded, resulting in 969 valid questionnaires for analysis. The effective rate of questionnaire was 83.61%. Our study was conducted in accordance with the Declaration of Helsinki and the protocol was approved by the Ethics Committee of Nanjing Jiangning Hospital.

### 2.2 Measurements

#### 2.2.1 Sugar-sweetened beverage intake

Sugar-sweetened beverage is generally defined as any beverage containing a caloric sweetener, whether as inherent in the product formula or added after purchase (33). A Chinese version of the Beverage Intake Questionnaire was developed to measure participants’ beverage intake. This questionnaire was modified from the updated version of Beverage Intake Questionnaire-15, which estimates calorie and volume intake of 15 beverages per day for adults (34). Considering China’s specific context, we categorized sugar-sweetened beverages into 100% fruit juice, 100% vegetable juice, sweetened fruit juice, sweet milk tea, sweetened tea drinks, carbonated drinks, artificially sweetened beverages, soda, whole milk and chocolate milk, low-fat milk, skim milk, soy milk, milk beverages, vegetable protein beverages, meal replacement shakes/protein drinks, coffee with milk and sugar, energy/sports drinks, spirits, wine, and beer. The Chinese version of the Beverage Intake Questionnaire demonstrated good internal consistency, with a Cronbach’s alpha coefficient of 0.937. Participants reported the frequency of beverage intake over the past month and the amount of each intake. The frequency was categorized as never or hardly ever, 1–3 times/month, 1 time/week, 2–3 times/week, 4–6 times/week, 1 time/day, 2 times/day, and 3 times/day or more, translating to the weekly frequency of 0, 0.5, 1, 2.5, 5, 7, 14, and 21, respectively. The amount per intake was divided into 7 categories: 150 mL, 250 mL, 350 mL, 450 mL, 550 mL, 650 mL, and 750 mL. The daily intake was determined by multiplying the weekly frequency and the amount per intake, and then dividing by 7 (35), using 250 mL as the standard serving size.

#### 2.2.2 Body esteem

The Body-Esteem Scale for Adolescents and Adults, developed by Mendelson et al. in 2001, was used to measure participants’ body esteem (16). This scale, comprising 23 items, was segmented into three subscales: body esteem-appearance (evaluating overall perceptions of appearance with 10 items), body esteem-attribution (assessing social evaluations attributed to one’s body and appearance with 5 items), and body esteem-weight (describing weight satisfaction with 8 items). Responses were recorded on a 5-point Likert scale, ranging from 0 (never) to 4 (always), generating a total score between 0 and 92. Nine items necessitated reverse scoring. Higher scores indicated elevated levels of body esteem and a positive body image (16). The reliability and validity of Body-Esteem Scale for Adolescents and Adults have been substantiated in various countries, including Indonesia (36), Brazil (37), and India (38). In our study, both Body-Esteem Scale for Adolescents and Adults and its three subscales demonstrated high internal consistency, with Cronbach’s *α* of 0.878 for overall, and 0.771, 0.831, and 0.852 for body esteem-appearance, body esteem-attribution, and body esteem-weight, respectively.

#### 2.2.3 Covariates

We identified several potential confounding variables, including gender, ethnicity, age, grade, major, body mass index (BMI), whether the participant was an only child, father’s educational level, mother’s educational level, living area, monthly family income, weight self-perception, ideal weight, and weight management behavior. To assess participants’ perceptions of their body image, we designed three questions taking the youth risk behavior surveillance system (39) on body weight as references. The first question asked participants to describe their weight perception, with responses categorized into three options: about the right weight, underweight, and overweight. The second question inquired about participants’ ideal weight, with responses categorized into three options: about the same as now, lighter than now, and heavier than now. Lastly, participants were asked about their weight management behavior, with responses coded into four categories: not trying to change weight, aiming to maintain current weight, attempting to lose weight, and endeavoring to gain weight.

### 2.3 Statistical analysis

First, participants’ daily intake of sugar-sweetened beverages was determined. Continuous variables were categorized based on the median into no or low sugar-sweetened beverage intake and high sugar-sweetened beverage intake. Continuous variables were described as mean and standard deviation (SD), and categorical variables were described as counts (n) and proportions (%). Comparisons between groups were conducted using *t*-tests for continuous variables and Chi-square tests for categorical variables. Second, three binary logistic regression models were established to assess the relationship between the three dimensions of body esteem and high sugar-sweetened beverage intake. Adjustment was made for statistically significant variables from univariate analysis, including gender, age, grade, father’s and mother’s educational levels, living area, and monthly family income. Based on previous studies (40–42), ethnicity, major, and BMI were included as potential confounding factors. Variance inflation factors were used to check for multicollinearity, with age selected as the independent variable due to a variance inflation factor value >2. Model 1 included three dimensions of body esteem. Model 2 was adjusted for gender, ethnicity, age, major, and BMI, while Model 3 further adjusted for whether the participant was an only child, the educational level of the parents, living area, and monthly family income. Results were expressed as odds ratios (ORs) and 95% confidence intervals (CIs). Third, restricted cubic spline plots were generated based on the results of linear regression to explore non-linear associations between variables. Subgroup analyses were conducted to explore the association between body esteem and high sugar-sweetened beverage intake across different subgroups and potential interactions. Subgroups were stratified by gender, ethnicity, age, major, BMI, one-child family, father’s educational level, mother’s educational level, living area, and monthly family income.

Statistical analyses and visualizations were conducted using SPSS 26.0 software (IBM Corp., Armonk, NY, United States) and R statistical package 4.2.2 (R Foundation for Statistical Computing, Vienna, Austria), with a two-sided *p*-value < 0.05 considered statistically significant.

## 3 Results

### 3.1 Study population characteristics

The characteristics of participants are summarized in Table 1. A total of 969 participants were included in the study, with 198 male individuals (20.4%) and 771 female individuals (79.6%). The mean age of participants was 20.07 years (SD = 1.65). Compared to individuals with non or low sugar-sweetened beverage intake, those with high sugar-sweetened beverage intake were more likely to be male, older, enrolled in higher grades, come from one-child families, have parents with higher educational levels, reside in cities or towns, possess relatively higher monthly family incomes, and score higher on the body esteem-attribution scale.

**Table 1:** Characteristics of participants stratified by sugar-sweetened beverage intake.

Characteristics	All (*n* = 969) N (%) or mean ± SD	Non or low sugar-sweetened beverage intake (*n* = 481) N (%) or mean ± SD	High sugar-sweetened beverage intake (*n* = 488) N (%) or mean ± SD	*p* value
Gender				<0.001
Male	198 (20.4)	69 (34.8)	129 (65.2)	
Female	771 (79.6)	412 (53.4)	359 (46.6)	
Ethnicity				0.051
Han	901 (93.0)	455 (50.5)	446 (49.5)	
Others	68 (7.0)	26 (38.2)	42 (61.8)	
Age (year)	20.07 ± 1.65	19.93 ± 1.63	20.21 ± 1.67	0.008
Grade				0.001
Grade 1	354 (36.5)	199 (56.2)	155 (43.8)	
Grade 2	152 (15.7)	59 (38.8)	93 (61.2)	
Grade 3	227 (23.4)	118 (52.0)	109 (48.0)	
Grade 4 and above	236 (24.4)	105 (44.5)	131 (55.5)	
Major				0.059
Non-medical specialty	507 (52.3)	237 (46.7)	270 (53.3)	
Medical specialty	462 (47.7)	244 (52.8)	218 (47.2)	
BMI (kg/m^2^)	20.73 ± 3.03	20.70 ± 2.87	20.79 ± 3.18	0.524
One-child family				0.006
No	511 (52.7)	275 (53.8)	236 (46.2)	
Yes	458 (47.3)	206 (45.0)	252 (55.0)	
Father’s educational level				<0.001
Primary school and below	106 (10.9)	58 (54.7)	48 (45.3)	
Junior high school	322 (33.2)	185 (57.5)	137 (42.5)	
Senior high school/technical secondary school	351 (36.2)	163 (46.4)	188 (53.6)	
College and above	190 (19.6)	75 (39.5)	115 (60.5)	
Mother’s educational level				0.015
Primary school and below	180 (18.6)	100 (55.6)	80 (44.4)	
Junior high school	319 (32.9)	168 (52.7)	151 (47.3)	
Senior high school/technical secondary school	306 (31.6)	148 (48.4)	158 (51.6)	
College and above	164 (16.9)	65 (39.6)	99 (60.4)	
Living area				<0.001
City	395 (40.8)	162 (41.0)	233 (59.0)	
Town	313 (32.3)	160 (51.1)	153 (48.9)	
Rural area	261 (26.9)	159 (60.9)	102 (39.1)	
Family income/monthly(yuan)				<0.001
<2,000	39 (4.0)	25 (64.1)	14 (35.9)	
2,000–4,999	209 (21.6)	123 (58.9)	86 (41.1)	
5,000–7,999	270 (27.9)	146 (54.1)	124 (45.9)	
≥8,000	451 (46.5)	187 (41.5)	264 (58.5)	
Weight self-perception				0.732
About the right weight	445 (45.9)	217 (48.8)	228 (51.2)	
Underweight	125 (12.9)	60 (48.0)	65 (52.0)	
Overweight	399 (41.2)	204 (51.1)	195 (48.9)	
Ideal weight				0.331
About the same as now	181 (18.7)	86 (47.5)	95 (52.5)	
Lighter than now	694 (71.6)	354 (51.0)	340 (49.0)	
Heavier than now	94 (9.7)	41 (43.6)	53 (56.4)	
Weight management behavior				0.123
I am not trying to do anything about my weight	186 (19.2)	104 (55.9)	82 (44.1)	
Stay the same weight	223 (23.0)	104 (46.6)	119 (53.4)	
Lose weight	505 (52.1)	251 (49.7)	254 (50.3)	
Gain weight	55 (5.7)	22 (40.0)	33 (60.0)	
Body esteem-appearance	22.60 ± 5.70	22.84 ± 5.69	22.36 ± 5.71	0.189
Body esteem-attribution	9.36 ± 3.75	8.88 ± 3.59	9.83 ± 3.86	<0.001
Body esteem-weight	16.21 ± 5.96	16.16 ± 5.95	16.26 ± 5.98	0.798

Chi-square test and *t*-test were applied in an equilibrium test between characteristics of non or low sugar-sweetened beverage intake participants and high sugar-sweetened beverage intake participants.

### 3.2 Body esteem scores across subgroups

The body esteem scores across subgroups are summarized in Table 2. Individuals with higher body esteem-appearance scores were more likely to possess relatively higher monthly family incomes, perceive the right weight, have ideal weight that the same as now, and not try to do anything about weight or stay the same weight. Individuals with higher body esteem-attribution scores were more likely to be enrolled in higher grades, underweight, have fathers with higher educational levels, reside in cities or towns, possess relatively higher monthly family incomes, perceive the right weight, have ideal weight that the same as now, and to gain weight or stay the same weight. Individuals with higher body esteem-weight scores were more likely to be underweight, perceive the right weight, have ideal weight that the same as now, and not to do anything about weight or stay the same weight.

**Table 2:** Body esteem scores across subgroups.

Characteristics	Body esteem-appearance Mean ± SD	*p* value	Body esteem-attribution Mean ± SD	*p* value	Body esteem-weight Mean ± SD	*p* value
Gender		0.169		0.419		0.055
Male	22.10 ± 5.93		9.17 ± 4.26		16.93 ± 6.04	
Female	22.73 ± 5.64		9.41 ± 3.62		16.02 ± 5.93	
Ethnicity		0.936		0.146		0.573
Han	22.60 ± 5.75		9.41 ± 3.74		16.18 ± 5.90	
Others	22.54 ± 4.97		8.72 ± 3.91		16.60 ± 6.74	
Grade		0.074		0.004		0.469
Grade 1	23.05 ± 5.98		8.82 ± 3.84		15.81 ± 6.31	
Grade 2	21.64 ± 5.86		9.31 ± 3.71		16.28 ± 5.95	
Grade 3	22.73 ± 4.99		9.72 ± 3.61		16.47 ± 5.29	
Grade 4 and above	22.40 ± 5.76		9.85 ± 3.71		16.51 ± 6.03	
Major		0.401		0.090		0.786
Non-medical specialty	22.45 ± 5.33		9.55 ± 3.59		16.16 ± 5.85	
Medical specialty	22.76 ± 6.09		9.15 ± 3.92		16.26 ± 6.08	
BMI		0.112		<0.001		<0.001
<18.5	23.02 ± 5.28		10.35 ± 3.61		19.92 ± 5.12	
18.5 ~ <24	22.68 ± 5.39		9.45 ± 3.59		15.96 ± 5.41	
≥24	21.34 ± 7.67		7.04 ± 3.95		10.72 ± 5.61	
One-child family		0.121		0.578		0.966
No	22.33 ± 5.58		9.30 ± 3.73		16.22 ± 5.85	
Yes	22.90 ± 5.82		9.43 ± 3.78		16.20 ± 6.08	
Father’s educational level		0.329		0.028		0.322
Primary school and below	22.34 ± 4.85		9.00 ± 3.70		16.08 ± 5.37	
Junior high school	22.20 ± 5.36		8.94 ± 3.38		15.93 ± 5.66	
Senior high school/technical secondary school	22.95 ± 5.67		9.69 ± 3.78		16.68 ± 5.85	
College and above	22.75 ± 6.66		9.66 ± 4.24		15.89 ± 6.89	
Mother’s educational level		0.471		0.059		0.527
Primary school and below	22.32 ± 5.31		8.77 ± 3.69		15.68 ± 5.77	
Junior high school	22.31 ± 5.36		9.30 ± 3.54		16.28 ± 5.56	
Senior high school/technical secondary school	22.86 ± 5.95		9.72 ± 3.73		16.50 ± 6.24	
College and above	22.96 ± 6.27		9.46 ± 4.20		16.10 ± 6.39	
Living area		0.223		<0.001		0.146
City	22.71 ± 5.75		9.88 ± 3.87		16.12 ± 6.46	
Town	22.88 ± 5.82		9.44 ± 3.67		16.68 ± 5.69	
Rural area	22.09 ± 5.46		8.47 ± 3.53		15.78 ± 5.44	
Family income/monthly(yuan)		0.020		<0.001		0.595
<5,000	21.77 ± 5.88		8.27 ± 3.87		15.93 ± 5.55	
5,000–7,999	22.65 ± 5.52		9.11 ± 3.61		16.14 ± 5.99	
≥8,000	23.02 ± 5.67		10.10 ± 3.62		16.40 ± 6.16	
Weight self-perception		<0.001		<0.001		<0.001
About the right weight	23.76 ± 5.17		10.26 ± 3.53		19.08 ± 4.75	
Underweight	22.36 ± 4.95		9.89 ± 3.62		19.07 ± 5.31	
Overweight	21.38 ± 6.21		8.19 ± 3.73		12.11 ± 4.88	
Ideal weight		0.022		<0.001		<0.001
About the same as now	23.53 ± 4.84		10.27 ± 3.61		21.61 ± 4.59	
Lighter than now	22.34 ± 5.95		9.05 ± 3.78		14.60 ± 5.43	
Heavier than now	22.68 ± 5.17		9.90 ± 3.53		17.68 ± 5.56	
Weight management behavior		<0.001		0.009		<0.001
I am not trying to do anything about my weight	23.64 ± 5.17		8.98 ± 3.54		19.29 ± 5.19	
Stay the same weight	23.56 ± 5.12		9.94 ± 3.55		19.95 ± 4.54	
Lose weight	21.86 ± 6.03		9.15 ± 3.86		13.39 ± 5.29	
Gain weight	21.98 ± 5.49		10.18 ± 3.93		16.55 ± 5.78	

*t-test and analysis of variance were applied to examine the variations in scores for body esteem-appearance, body esteem-attribution and body esteem-weight across subgroups.*

### 3.3 Association between body esteem and high sugar-sweetened beverage intake using logistic model

Results of logistic regression models examining the association between body esteem and the risk of high sugar-sweetened beverage intake are presented in Table 3. Model 1 incorporated three dimensions of body esteem. Body esteem-appearance was negatively associated with high sugar-sweetened beverage intake; a one-unit increase in body esteem-appearance score corresponded to a 3.9% decrease in the likelihood of high sugar-sweetened beverage intake (OR = 0.961, 95%CI = 0.936–0.987, *p* = 0.003). Conversely, body esteem-attribution was positively associated with high sugar-sweetened beverage intake; a one-unit increase in body esteem-attribution score correlated with a 10.2% increase in the likelihood of high sugar-sweetened beverage intake (OR = 1.102, 95%CI = 1.059–1.146, *p* < 0.001). Upon adjusting for individual-related variables in Model 2, including gender, ethnicity, age, major, and BMI, the associations persisted. Specifically, body esteem-appearance was negatively associated with high sugar-sweetened beverage intake (OR = 0.965, 95%CI = 0.939–0.992, *p* = 0.011), while body esteem-attribution was positively associated with high sugar-sweetened beverage intake (OR = 1.112, 95%CI = 1.067–1.159, *p* < 0.001). These associations remained significant even after further adjustments for household-related variables in Model 3, including whether the participant was an only child, parents’ educational level, living area, and monthly family income. Specifically, body esteem-appearance was negatively associated with high sugar-sweetened beverage intake (OR = 0.962, 95%CI = 0.935–0.989, *p* = 0.007), while body esteem-attribution was positively associated with high sugar-sweetened beverage intake (OR = 1.091, 95%CI = 1.046–1.139, *p* < 0.001). No significant association was observed between body esteem-weight and high sugar-sweetened beverage intake across all three models (*p* > 0.05).

**Table 3:** Association between body esteem and high sugar-sweetened beverage intake.

Model	Body esteem-appearance		Body esteem-attribution		Body esteem-weight	
	OR (95%CI)	*p* value	OR (95%CI)	*p* value	OR (95%CI)	*p* value
Model 1^a^	0.961 (0.936, 0.987)	0.003	1.102 (1.059, 1.146)	<0.001	0.996 (0.972, 1.021)	0.757
Model 2^b^	0.965 (0.939, 0.992)	0.011	1.112 (1.067, 1.159)	<0.001	0.991 (0.963, 1.019)	0.520
Model 3^c^	0.962 (0.935, 0.989)	0.007	1.091 (1.046, 1.139)	<0.001	0.996 (0.968, 1.025)	0.781

a Model 1 included body esteem-appearance, body esteem-attribution and body esteem-weight.

b Model 2 was adjusted for gender, ethnicity, age, major and BMI.

c Model 3 was additionally adjusted for one-child family, father’s educational level, mother’s educational level, living area and monthly family income.

OR, odds ratio; CI, confidence interval.

### 3.4 Investigation of non-linear association using restricted cubic spline

Restricted cubic spline plots were used to explore potential non-linear associations between the three dimensions of body esteem and high sugar-sweetened beverage intake, while controlling for all confounding factors (all covariates in Model 3).

**Figure 1:**
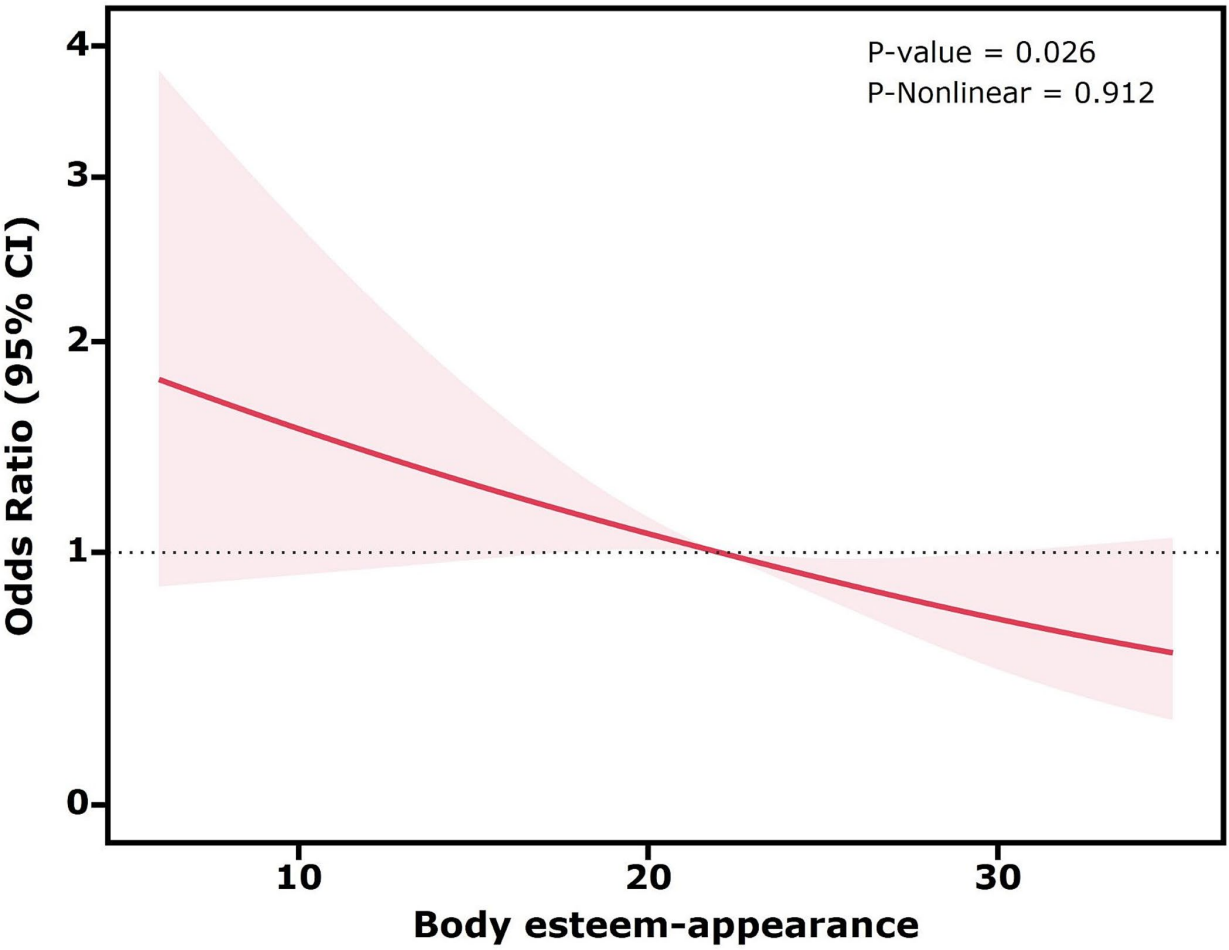
Restricted cubic spline analysis of the association between body esteem-appearance and high sugar-sweetened beverage intake.

**Figure 2:**
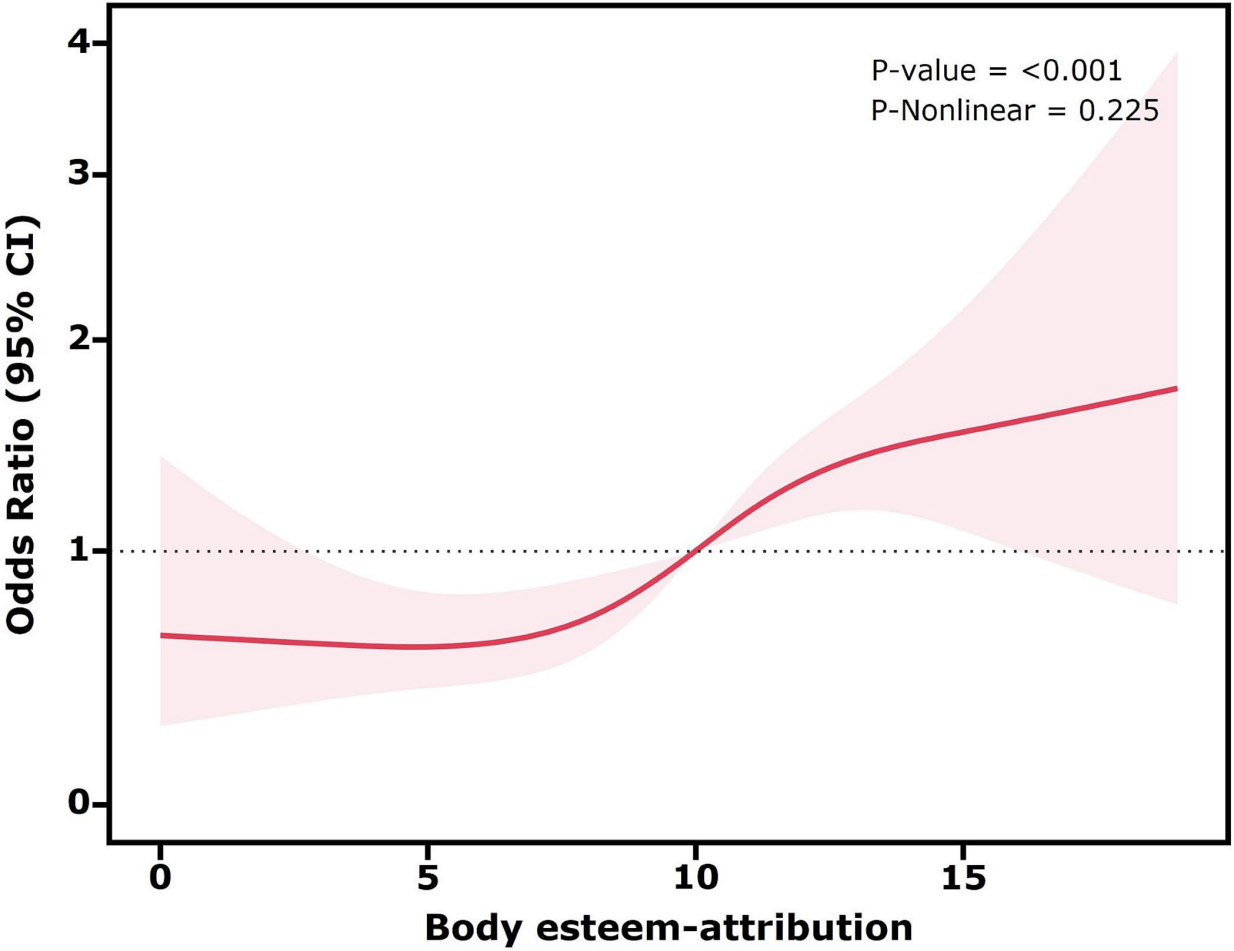
Restricted cubic spline analysis of the association between body esteem-attribution and high sugar-sweetened beverage intake.

**Figure 3:**
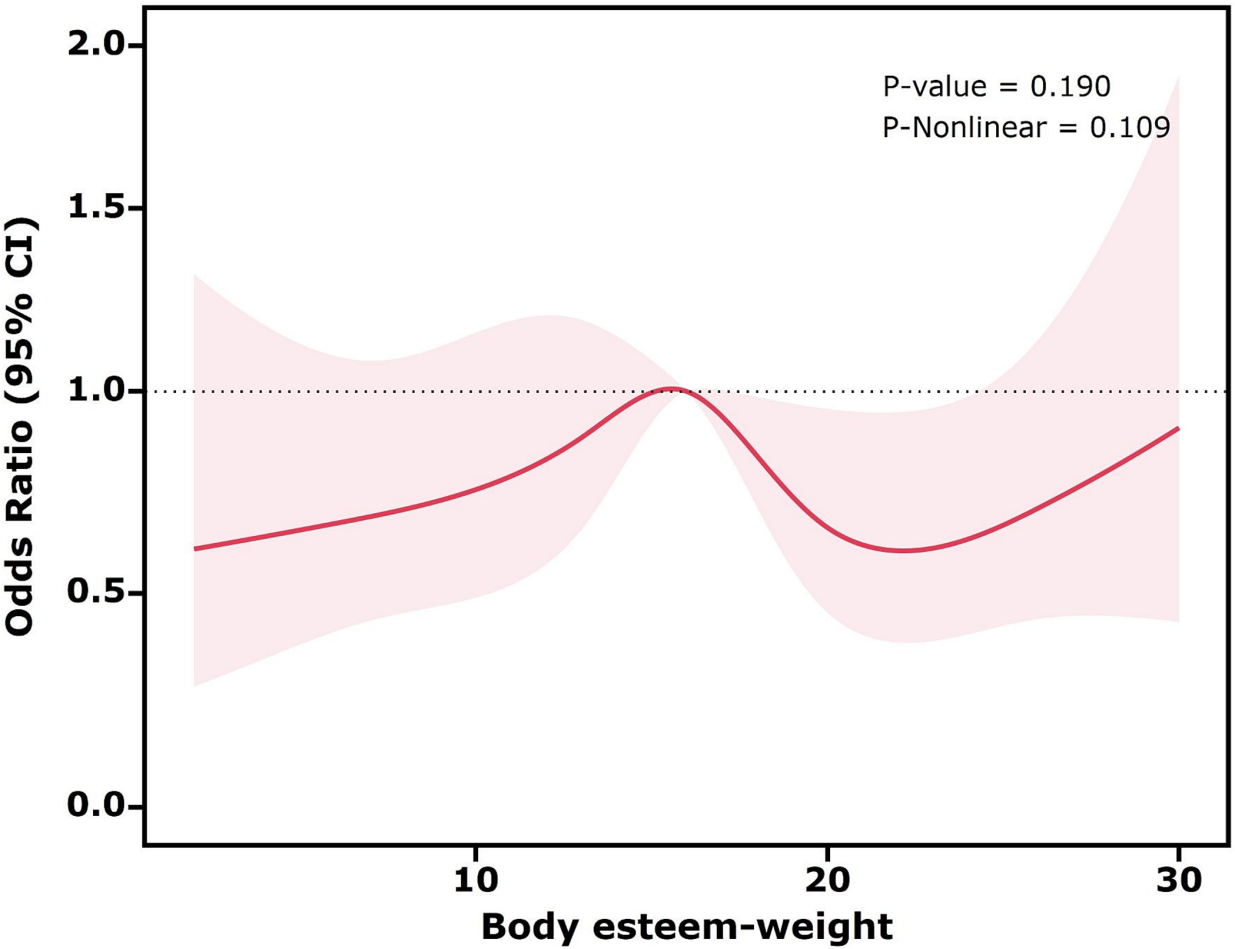
Restricted cubic spline analysis of the association between body esteem-weight and high sugar-sweetened beverage intake.

As shown in Figure 1, the *p*-value for the non-linearity test was 0.912, suggesting no significant non-linear association between body esteem-appearance and high sugar-sweetened beverage intake. The *p*-value was 0.026, and the decreasing trend observed was approximately linear, indicating a negative association between body esteem-appearance and high sugar-sweetened beverage intake. Similarly, as shown in Figure 2, the *p*-value for the non-linearity test was 0.225, indicating no significant non-linear association between body esteem-attribution and high sugar-sweetened beverage intake. The *p*-value < 0.001 and the overall upward trend indicated a positive association between body esteem-attribution and high sugar-sweetened beverage intake. Conversely, Figure 3 depicts the relationship between body esteem-weight and high sugar-sweetened beverage intake. Both the non-linearity test *p*-value and the *p*-value were greater than 0.05, indicating no significant association between the two variables.

### 3.5 Subgroup and interaction analyses

**Figure 4:**
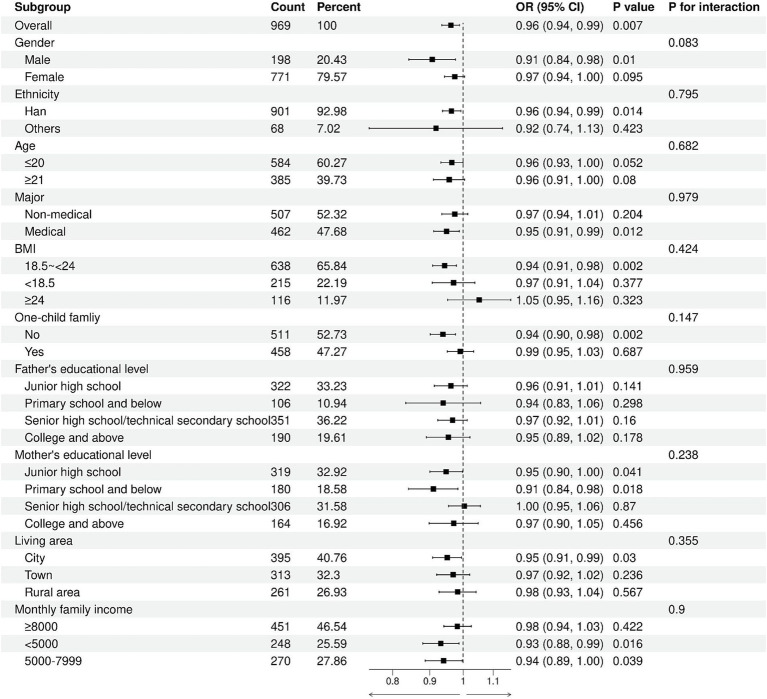
Subgroup analysis of the association of body esteem-appearance with high sugar-sweetened beverage intake.

**Figure 5:**
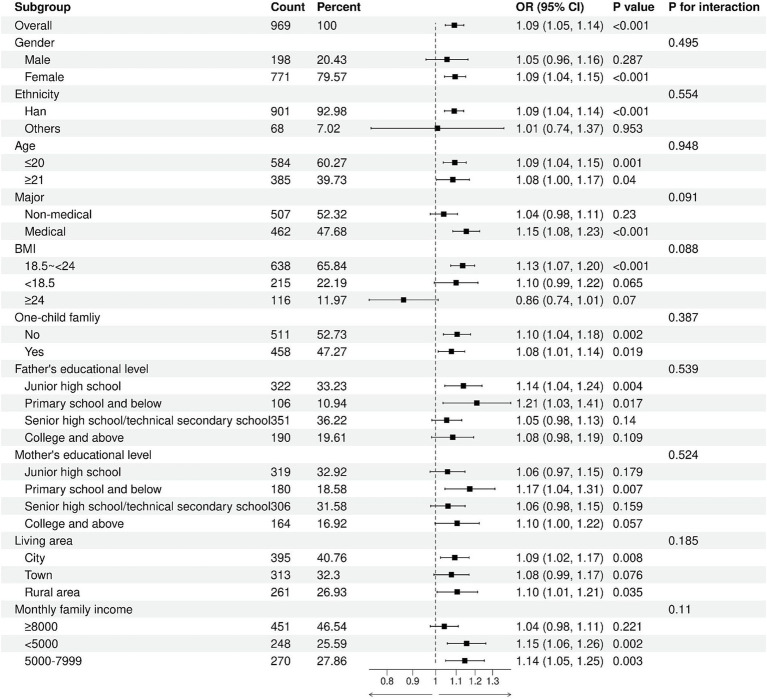
Subgroup analysis of the association of body esteem-attribution with high sugar-sweetened beverage intake.

No significant interaction was observed between body esteem-appearance and each subgroup (*p* for interaction: 0.083–0.979), suggesting that the association between body esteem-appearance and the risk of high sugar-sweetened beverage intake was independent and not influenced by other covariates (Figure 4). Likewise, no significant interaction was found between body esteem-attribution and each subgroup (*p* for interaction: 0.088–0.948), indicating that the association between body esteem-attribution and the risk of high sugar-sweetened beverage intake was independent and consistent across subgroups (Figure 5).

## 4 Discussion

In this cross-sectional study, we examined the association between body esteem and the risk of high sugar-sweetened beverage intake. To the best of our knowledge, this is the first study to explore the association of the three dimensions of body esteem and sugar-sweetened beverage intake among undergraduate students. Our findings revealed that in fully adjusted multivariate analyses, there was a negative association between body esteem-appearance and the risk of high sugar-sweetened beverage intake, whereas body esteem-attribution displayed a positive association with the risk of high sugar-sweetened beverage intake. However, no significant association was observed between body esteem-weight and the risk of high sugar-sweetened beverage intake among Chinese undergraduate students. Further analyses, including non-linear tests and subgroup analyses, further confirmed these findings. This study was conducted in a medical university, where nutrition knowledge is generally insufficient even among medical students in China (6). In addition, this study included both medical and non-medical undergraduate students, its findings were applicable to other undergraduate populations as well.

Our study revealed that Chinese undergraduate students had an average sugar-sweetened beverage intake of 446.53 mL, equivalent to approximately 1.8 standard servings. Unlike previous studies which solely reported the frequency of sugar-sweetened beverage intake among Chinese university students, our study uniquely quantified the total amount consumed. Among Chinese Tibetan college students, proportions of those consuming sugar-sweetened beverages ≤1 time per week, 2–5 times per week, and ≥6 times per week were 54.2, 24.3, and 21.5%, respectively (43). Similarly, a study of college freshmen in China showed that over half of the students consumed sugar-sweetened beverages more than three times a week (44). Comparing sugar-sweetened beverage intake with young adults abroad, Saudi undergraduate students had a daily intake of 650.60 mL, while Brazilian young adults consumed 281.5 mL (45, 46). However, this comparison is somewhat limited due to variations in sugar-sweetened beverage definitions and included beverage categories across different countries. Furthermore, since most of the participants in our study were female, this may result in an underestimation of sugar-sweetened beverage intake. Our study showed that the beverages with high consumption among Chinese undergraduate students were sweet milk tea, sweetened tea drinks, sweetened fruit juice and different types of milk, generally consistent with previous studies in Chinese college populations (8). In contrast, studies in other countries revealed different popular beverages; for instance, fruit juices, soda, artificially sweetened soda, sports, and energy drinks were popular in Australia (47), while hot drinks, fruit drinks, and soda were favored among Jordanian university students (27). These findings suggest the differences in beverage consumption patterns between China and the Western regions.

Our study indicated that individuals with high sugar-sweetened beverage intake were more likely to be male. Given that the average weight of males is higher than that of females, male individuals generally consume more energy than females (48). Individuals with high sugar-sweetened beverage intake were more likely to come from higher grades. With the increase of enrollment time, undergraduate students may increasingly be exposed to beverages such as alcohol and coffee. In addition, the association between family and sugar-sweetened beverage intake has been confirmed in previous studies (49). In China, families with only one-child and parents with higher educational levels typically also have higher family incomes, enabling students to afford more beverages. Our study showed that individuals with high sugar-sweetened beverage intake were more likely to reside in cities or towns, which was inconsistent with studies in Canada (50). This discrepancy may be due to the significant rural–urban divide in China, where convenience stores that sell beverages like milk tea and coffee are concentrated in cities and urban areas. Our study revealed that individuals with higher body esteem scores were more likely to perceive the right weight, have ideal weight that the same as now, not try to do anything about weight, stay the same weight or gain weight. This may be because those who perceive themselves as “normal weight” internalized weight stigma less than those who perceive themselves as “normal weight,” leading to higher body satisfaction (51). Body esteem-attribution and body esteem-weight were somewhat linked to BMI, suggesting that external evaluations are influenced by degree of thinness and fatness, prompting individuals to pay attention to their body shapes. Furthermore, body esteem-attribution is related to grade, father’s educational level, place of residence, and family incomes. Compared to the other dimensions of body esteem, body esteem-attribution, which is influenced by external evaluations, has more influencing factors and requires attention.

The negative association observed between body esteem-appearance and the risk of high sugar-sweetened beverage intake among Chinese undergraduate students suggests that individuals more satisfied with their appearances were less likely to consume sugar-sweetened beverages. According to health belief model, the association between body esteem-appearance and the risk of high sugar-sweetened beverage intake may be influenced by individuals’ attitudes and perceptions toward sugar-sweetened beverages. Health belief model analyzes how personal beliefs influence behaviors by assessing the benefits and potential consequences of preventive behaviors (52). With advancements in modern medicine, health belief model has expanded beyond traditional disease-related behaviors to include general health behaviors such as diet (53) and vaccination (54), and also attitudes toward sugar-sweetened beverages. Health belief model consists of four components: perceived susceptibility, perceived severity, perceived benefits of taking health actions, and perceived barriers (55). Undergraduate students with high levels of body esteem-appearance often focus more on health issues related to body image, such as obesity and weight gain (3). Since sugar-sweetened beverages are associated with weight gain and obesity, these students are more likely to perceive themselves as vulnerable to negative effects of sugar-sweetened beverages. This means they have higher perceived susceptibility to health problems caused by sugar-sweetened beverages and stronger perceptions of severity of these issues. Reducing sugar-sweetened beverage intake can be seen a way to lower the risk of weight gain, maintain or improve body shape, and enhance body image. Positive expectations of health outcomes encourage them to develop favorable attitudes toward reducing sugar-sweetened beverage intake. For undergraduate students with high levels of body esteem-appearance, perceived barriers can be viewed as “psychological cost” of sugar-sweetened beverage intake, as it may threaten their ideal body images and thus increase psychological burden and social pressure. Consequently, these students are likely to choose to reduce sugar-sweetened beverage intake to avoid these “psychological costs.”

The positive association observed between body esteem-attribution and the risk of high sugar-sweetened beverage intake among Chinese undergraduate students suggests that individuals who are less satisfied with their appearances or bodies, as evaluated externally, tend to consume fewer sugar-sweetened beverages. Previous study has revealed that others’ evaluations of individuals’ bodies can significantly influence their intuitive eating habits. Specifically, the more negative evaluations, the lower levels of intuitive eating (56). Intuitive eating is an eating approach that relies on internal cues such as hunger and fullness. It encourages individuals to select foods based on bodily needs and preferences, rather than being swayed by external pressures like social expectations, food trends, or emotional states (57). The sweet components present in sugar-sweetened beverages are known as to evoke feelings of happiness and indulgence (58), making sugar-sweetened beverage intake a manifestation of intuitive eating. However, when individuals perceive others’ evaluations of their appearances or bodies to be negative, it can hinder their natural urge to enjoy sugar-sweetened beverages as part of their intuitive eating practice. According to self-construct theory, cultural differences play a pivotal role in shaping individuals’ self-identity. Eastern cultures, characterized by collectivism, prioritize social acceptance, whereas Western cultures, rooted in individualism, emphasized personal autonomy (59). Previous study indicated that individuals from Eastern backgrounds often base self-worth on acceptance or rejection received from communities (60). Consequently, external evaluations about their appearances or bodies can profoundly influence self-perceptions (61). In pursuit of idealized body images and desire to garner positive feedback from others, Chinese undergraduate students may actively regulate sugar-sweetened beverage intake.

There was no significant association found between body esteem-weight and the risk of high sugar-sweetened beverage intake among Chinese undergraduate students. Previous study found no significant association between wight self-perception and sugar-sweetened beverage intake among adolescents in New Caledonia, aligning with our results (62). The study suggested that effective weight management involves not only controlling sugar-sweetened beverage intake but also managing other energy-dense foods intake (62). Since our study did not assess total energy intake, this could explain the lack of significant association between body esteem-weight and sugar-sweetened beverage intake. More likely, the findings reflect tendencies to be lenient about their weight among some undergraduate students. Despite engaging in weight management practices such as exercise and dieting, college students exhibited poor outcomes and low adherence to weight management behaviors due to insufficient resolve and motivation (63). Consequently, when considering sugar-sweetened beverage intake as a dietary behavior, undergraduate students may struggle to develop effective diet plans and implement them. In addition, studies have shown that when individuals view their bodies as similar to those of others who do not meet societal ideals, they may experience strong feelings of embarrassment and shame, which can lead to responses of disposal or separation (64). Unlike products that can be discarded, donated, or sold, individuals’ bodies cannot be managed in the same way, potentially resulting in resignation (65). Some undergraduate students with low weight satisfaction may successfully control sugar-sweetened beverage intake through weight management, while others who struggle with weight management might have difficulty controlling sugar-sweetened beverage intake. Furthermore, some students may resort to emotional eating and excessive sugar-sweetened beverage intake when faced with poor weight satisfaction. This polarization in behavior is a key reason resulting in the lack of a significant association between body esteem-weight and sugar-sweetened beverage intake.

This study broadens the dimension of self-determination theory’s psychological needs, positing that body esteem as a psychological need that merits comprehensive exploration. It further supports the validity of self-determination theory and its applicability in the field of dietary behaviors. The results of this study also provide managerial implications for improving eating behaviors of undergraduate students. On the one hand, from the perspective of self-management, undergraduate students need to cultivate a correct perception of their appearance, adopt appropriate image management strategies, and enhance their appearance satisfaction, thereby constructing rational dietary behaviors. On the other hand, from the perspective of external management, a healthy and uplifting esthetic and dietary perception should be collectively fostered by friends, faculty, and media, effectively guiding students to adopt sound esthetic and dietary practices. Universities should consistently organize sports activities and health initiatives, fostering vibrant and positive campus culture. By conducting professional nutrition education lectures and considering the unique needs of each student, personalized health management plans should be crafted. To amplify influence, schools should leverage diverse promotional channels, including posters and online media, to disseminate correct esthetic concepts and healthy nutritional knowledge, with the objective of effectively boosting students’ body esteem and reducing sugar-sweetened beverage intake.

However, there were some limitations to our study. Firstly, it was a cross-sectional study and therefore causality could not be established. Longitudinal studies should be carried out in future to elucidate the causal relationship between the three dimensions of body esteem and the risk of high sugar-sweetened beverage intake. Secondly, due to convenience sampling, our data were obtained from a single medical university in China with a higher proportion of female participants, limiting the generalizability of the findings. Random sampling methods for multi-center studies should be conducted across various types of universities. Thirdly, our study relied on self-reported data from participants, which may be influenced by recall bias and expectation bias, necessitating cautious interpretation of the results. Lastly, despite controlling numerous confounding variables, the presence of residual confounding bias cannot be ruled out, as certain potential characteristics may not have been considered.

## 5 Conclusion

In summary, our study revealed a negative association between body esteem-appearance and the risk of high sugar-sweetened beverage intake, as well as a positive association between body esteem-attribution and the risk of high sugar-sweetened beverage intake among Chinese undergraduate students. It indicated that undergraduates who were more satisfied with their appearances were less prone to high sugar-sweetened beverage intake. Additionally, the worse the external evaluations of individuals’ appearances, the less susceptible to high sugar-sweetened beverage intake. These findings suggest that educational institutions or governmental bodies should offer both individual and group counseling, organize supportive campus activities, and provide self-affirmation training, aimed at helping students develop positive body images and steering undergraduate beverage intake toward healthier patterns. Nevertheless, given the limitations of our study, further well-designed longitudinal and multi-center studies are warranted to validate these findings.

## Data Availability

The raw data supporting the conclusions of this article will be made available by the authors, without undue reservation.

## Footnotes

^1^ http://www.wjx.cn

## References

[1] World Health Organization. Guideline: sugars intake for adults and children. (2015). Available at: https://iris.who.int/handle/10665/149782 (Accessed May 29, 2024). 25905159

[2] Chinese Nutrition Society. Dietary guidelines for Chinese residents. Beijing: Peoples Health Publishing House (2022).

[3] Malik VS Hu FB. The role of sugar-sweetened beverages in the global epidemics of obesity and chronic diseases. Nat Rev Endocrinol. (2022) 18:205–18. doi: 10.1038/s41574-021-00627-6, PMID: 35064240 PMC8778490

[4] Wu M Xi Y Huo J Xiang C Yong C Liang J. Association between eating habits and sodium intake among Chinese university students. Nutrients. (2023) 15:1570. doi: 10.3390/nu15071570, PMID: 37049412 PMC10097125

[5] Liu H Yang Q Luo J Ouyang Y Sun M Xi Y. Association between emotional eating, depressive symptoms and laryngopharyngeal reflux symptoms in college students: a cross-sectional study in Hunan. Nutrients. (2020) 12:1595. doi: 10.3390/nu12061595, PMID: 32485841 PMC7352624

[6] Liu S Wang J He G Chen B Jia Y. Evaluation of dietary quality based on intelligent ordering system and Chinese healthy eating index in college students from a medical School in Shanghai, China. Nutrients. (2022) 14:1012. doi: 10.3390/nu14051012, PMID: 35267987 PMC8912503

[7] Wang Y Bi C Liu H Lin H Cai R Zhang J. Association of sugar-sweetened beverage consumption with psychological symptoms among Chinese university students during the COVID-19 pandemic. Front Psychol. (2022) 13:1024946. doi: 10.3389/fpsyg.2022.1024946, PMID: 36312111 PMC9608563

[8] Xu H Yang Z Liu D Yu C Zhao Y Yang J. Mediating effect of physical sub-health in the association of sugar-sweetened beverages consumption with depressive symptoms in Chinese college students: a structural equation model. J Affect Disord. (2023) 342:157–65. doi: 10.1016/j.jad.2023.09.020, PMID: 37730148

[9] Haynes-Maslow L Ray S Giombi K. Perceptions of sugar-sweetened beverages among adolescents in North Carolina. Front Public Health. (2022) 10:943295. doi: 10.3389/fpubh.2022.943295, PMID: 36249251 PMC9557148

[10] Qin P Li Q Zhao Y Chen Q Sun X Liu Y. Sugar and artificially sweetened beverages and risk of obesity, type 2 diabetes mellitus, hypertension, and all-cause mortality: a dose-response meta-analysis of prospective cohort studies. Eur J Epidemiol. (2020) 35:655–71. doi: 10.1007/s10654-020-00655-y, PMID: 32529512

[11] Hu D Cheng L Jiang W. Sugar-sweetened beverages consumption and the risk of depression: a meta-analysis of observational studies. J Affect Disord. (2019) 245:348–55. doi: 10.1016/j.jad.2018.11.015, PMID: 30419536

[12] Kleppang AL de Ridder K Haugland SH Stea TH. Physical activity, sugar-sweetened beverages, whole grain bread and insomnia among adolescents and psychological distress in adulthood: prospective data from the population-based HUNT study. Int J Behav Nutr Phys Act. (2021) 18:143. doi: 10.1186/s12966-021-01215-7, PMID: 34724961 PMC8559387

[13] Mayer J Verhoeven AAC Dornic Q Hanzouli H Seksek I Guelinckx I. Understanding attitudes to change to healthier hydration habits: the case of high sugar: low water drinkers in Mexico. Ann Nutr Metab. (2020) 76:43–52. doi: 10.1159/000515023, PMID: 33774611

[14] Miller C Dono J Wright K Pettigrew S Wakefield M Coveney J. “No child or adult would ever probably choose to have 16 teaspoons of sugar”: a preliminary study of parents’ responses to sugary drink warning label options. Nutrients. (2022) 14:4173. doi: 10.3390/nu14194173, PMID: 36235825 PMC9571345

[15] Raspovic A Prichard I Salim A Yager Z Hart L. Body image profiles combining body shame, body appreciation and body mass index differentiate dietary restraint and exercise amount in women. Body Image. (2023) 46:117–22. doi: 10.1016/j.bodyim.2023.05.007, PMID: 37290141

[16] Mendelson BK Mendelson MJ White DR. Body-esteem scale for adolescents and adults. J Pers Assess. (2001) 76:90–106. doi: 10.1207/S15327752JPA7601_6, PMID: 11206302

[17] Zartaloudi A Christopoulos D Kelesi M Govina O Mantzorou M Adamakidou T. Body image, social physique anxiety levels and self-esteem among adults participating in physical activity programs. Dis Basel Switz. (2023) 11:66. doi: 10.3390/diseases11020066, PMID: 37218879 PMC10204469

[18] Sharif-Nia H Sivarajan Froelicher E Gorgulu O Osborne JW Błachnio A Rezazadeh Fazeli A. The relationship among positive body image, body esteem, and eating attitude in Iranian population. Front Psychol. (2024) 15:1304555. doi: 10.3389/fpsyg.2024.1304555, PMID: 38434953 PMC10905648

[19] Carbonneau N Holding A Lavigne G Robitaille J. Feel good, eat better: the role of self-compassion and body esteem in mothers’ healthy eating Behaviours. Nutrients. (2021) 13:3907. doi: 10.3390/nu13113907, PMID: 34836162 PMC8625178

[20] Pelc A Winiarska M Polak-Szczybyło E Godula J Stępień AE. Low self-esteem and life satisfaction as a significant risk factor for eating disorders among adolescents. Nutrients. (2023) 15:1603. doi: 10.3390/nu15071603, PMID: 37049444 PMC10096620

[21] Pokhrel P Bennett BL Boushey CJ. Body esteem, weight-control outcome expectancies, and e-cigarette use among young adults. Nicotine Tob Res Off J Soc Res Nicotine Tob. (2021) 23:454–61. doi: 10.1093/ntr/ntaa009, PMID: 31927589 PMC7885785

[22] Prentice M Jayawickreme E Fleeson W. Integrating whole trait theory and self-determination theory. J Pers. (2019) 87:56–69. doi: 10.1111/jopy.12417, PMID: 29999534

[23] Oumrait NG Daivadanam M Absetz P Guwatudde D Berggreen-Clausen A Alvesson HM. Can self-determination explain dietary patterns among adults at risk of or with type 2 diabetes? A cross-sectional study in socio-economically disadvantaged areas in Stockholm. Nutrients. (2020) 12:620. doi: 10.3390/nu12030620, PMID: 32120791 PMC7146106

[24] Guertin C Barbeau K Pelletier L. Examining fat talk and self-compassion as distinct motivational processes in women’s eating regulation: a self-determination theory perspective. J Health Psychol. (2020) 25:1965–77. doi: 10.1177/1359105318781943, PMID: 29944012

[25] Koponen AM Simonsen N Suominen S. How to promote fruits, vegetables, and berries intake among patients with type 2 diabetes in primary care? A self-determination theory perspective. Health Psychol Open. (2019) 6:2055102919854977. doi: 10.1177/2055102919854977, PMID: 31218074 PMC6563407

[26] Wang X Lu C Niu L. Body image construction and mental health levels among college students: a data survey of Chinese university students. Front Public Health. (2023) 11:1268775. doi: 10.3389/fpubh.2023.1268775, PMID: 37869184 PMC10585169

[27] Bawadi H Khataybeh T Obeidat B Kerkadi A Tayyem R Banks AD. Sugar-sweetened beverages contribute significantly to college students’ daily caloric intake in Jordan: soft drinks are not the major contributor. Nutrients. (2019) 11:1058. doi: 10.3390/nu11051058, PMID: 31083526 PMC6566441

[28] Godos J Grosso G Castellano S Galvano F Caraci F Ferri R. Association between diet and sleep quality: a systematic review. Sleep Med Rev. (2021) 57:101430. doi: 10.1016/j.smrv.2021.101430, PMID: 33549913

[29] Kosendiak AA Adamczak BB Kuźnik Z Makles S. How dietary choices and nutritional knowledge relate to eating disorders and body esteem of medical students? A single-center cross-sectional study. Nutrients. (2024) 16:1414. doi: 10.3390/nu16101414, PMID: 38794652 PMC11123669

[30] Hayes JF Balantekin KN Graham AK Strube MJ Bickel WK Wilfley DE. Implementation intentions for weight loss in college students with overweight and obesity: a proof-of-concept randomized controlled trial. Transl Behav Med. (2021) 11:359–68. doi: 10.1093/tbm/ibaa038, PMID: 32359068 PMC7963295

[31] Kang H. Sample size determination and power analysis using the G*power software. J Educ Eval Health Prof. (2021) 18:17. doi: 10.3352/jeehp.2021.18.17, PMID: 34325496 PMC8441096

[32] Morgan JF Reid F Lacey JH. The SCOFF questionnaire: assessment of a new screening tool for eating disorders. BMJ. (1999) 319:1467–8. doi: 10.1136/bmj.319.7223.1467, PMID: 10582927 PMC28290

[33] Miller PE McKinnon RA Krebs-Smith SM Subar AF Chriqui J Kahle L. Sugar-sweetened beverage consumption in the U.S.: novel assessment methodology. Am J Prev Med. (2013) 45:416–21. doi: 10.1016/j.amepre.2013.05.014, PMID: 24050417

[34] Fausnacht AG Myers EA Hess EL Davy BM Hedrick VE. Update of the BEVQ-15, a beverage intake questionnaire for habitual beverage intake for adults: determining comparative validity and reproducibility. J Hum Nutr Diet Off J Br Diet Assoc. (2020) 33:729–37. doi: 10.1111/jhn.12749, PMID: 32283572

[35] Gan WY Mohamed SF Law LS. Unhealthy lifestyle associated with higher intake of sugar-sweetened beverages among Malaysian school-aged adolescents. Int J Environ Res Public Health. (2019) 16:2785. doi: 10.3390/ijerph16152785, PMID: 31382672 PMC6696103

[36] Garbett KM Craddock N Haywood S Hayes C Nasution K Saraswati LA. Translation and validation of the body esteem scale in adults and adolescents among Indonesian adolescents. Body Image. (2024) 48:101679. doi: 10.1016/j.bodyim.2024.101679, PMID: 38281340

[37] Smith HG Garbett KM Matheson EL Amaral ACS Meireles JFF Almeida MC. The body esteem scale for adults and adolescents: translation, adaptation and psychometric validation among Brazilian adolescents. Body Image. (2022) 42:213–21. doi: 10.1016/j.bodyim.2022.05.012, PMID: 35779360

[38] Garbett KM Lewis-Smith H Chaudhry A Uglik-Marucha N Vitoratou S Shroff H. Cultural adaptation and validation of the body esteem scale for adults and adolescents for use in English among adolescents in urban India. Body Image. (2021) 37:246–54. doi: 10.1016/j.bodyim.2021.02.012, PMID: 33743264

[39] Centers for Disease Control and Prevention.2021 state and local youth risk behavior survey. (2021). Available at: https://www.cdc.gov/yrbs/files/2021/pdf/2021-yrbs-standard-hs-questionnaire.pdf (Accessed May 29, 2024).

[40] Russo RG Northridge ME Wu B Yi SS. Characterizing sugar-sweetened beverage consumption for US children and adolescents by race/ethnicity. J Racial Ethn Health Disparities. (2020) 7:1100–16. doi: 10.1007/s40615-020-00733-7, PMID: 32152835 PMC7483241

[41] Sanne I Bjørke-Monsen A-L. Dietary behaviors and attitudes among Norwegian medical students. BMC Med Educ. (2023) 23:220. doi: 10.1186/s12909-023-04194-4, PMID: 37024871 PMC10080805

[42] Nguyen M Jarvis SE Tinajero MG Yu J Chiavaroli L Mejia SB. Sugar-sweetened beverage consumption and weight gain in children and adults: a systematic review and meta-analysis of prospective cohort studies and randomized controlled trials. Am J Clin Nutr. (2023) 117:160–74. doi: 10.1016/j.ajcnut.2022.11.008, PMID: 36789935

[43] Song W Su F Li S Song Y Chai G. Association between sugar-sweetened beverages and duration of physical exercise with psychological symptoms among Tibetan university students at high altitude. Front Psychol. (2024) 15:1380893. doi: 10.3389/fpsyg.2024.1380893, PMID: 38725953 PMC11079124

[44] Gao C Sun Y Zhang F Zhou F Dong C Ke Z. Prevalence and correlates of lifestyle behavior, anxiety and depression in Chinese college freshman: a cross-sectional survey. Int J Nurs Sci. (2021) 8:347–53. doi: 10.1016/j.ijnss.2021.05.013, PMID: 34307785 PMC8283720

[45] Islam MA Al-Karasneh AF Hussain AB Muhanna A Albu-Hulayqah T Naqvi AA. Assessment of beverage consumption by young adults in Saudi Arabia. Saudi Pharm J SPJ Off Publ Saudi Pharm Soc. (2020) 28:1635–47. doi: 10.1016/j.jsps.2020.10.010, PMID: 33424256 PMC7783230

[46] Bragança MLBM Bogea EG de Almeida Fonseca Viola PC Dos Santos Vaz J Confortin SC AMB M. High consumption of sugar-sweetened beverages is associated with low bone mineral density in young people: the Brazilian birth cohort consortium. Nutrients. (2023) 15:324. doi: 10.3390/nu15020324, PMID: 36678194 PMC9867470

[47] Miller C Ettridge K Wakefield M Pettigrew S Coveney J Roder D. Consumption of sugar-sweetened beverages, juice, artificially-sweetened soda and bottled water: an Australian population study. Nutrients. (2020) 12:817. doi: 10.3390/nu12030817, PMID: 32204487 PMC7146120

[48] Fontes AS Pallottini AC Vieira DADS Fontanelli M Marchioni D Cesar C. Demographic, socioeconomic and lifestyle factors associated with sugar-sweetened beverage intake: a population-based study. J Epidemiol. (2020) 23:e200003. doi: 10.1590/1980-549720200003, PMID: 32130392

[49] Warren C Hobin E Manuel DG Anderson LN Hammond D Jessri M. Socioeconomic position and consumption of sugary drinks, sugar-sweetened beverages and 100% juice among Canadians: a cross-sectional analysis of the 2015 Canadian community health survey-nutrition. Can J Public Health Rev Can Santé Publique. (2022) 113:341–62. doi: 10.17269/s41997-021-00602-8, PMID: 35138596 PMC9043056

[50] Button BLG McEachern LW Martin G Gilliland JA. Intake of fruits, vegetables, and sugar-sweetened beverages among a sample of children in rural northern Ontario, Canada. Child Basel Switz. (2022) 9:1028. doi: 10.3390/children9071028, PMID: 35884012 PMC9320505

[51] Lucibello KM Nesbitt AE Solomon-Krakus S Sabiston CM. Internalized weight stigma and the relationship between weight perception and negative body-related self-conscious emotions. Body Image. (2021) 37:84–8. doi: 10.1016/j.bodyim.2021.01.010, PMID: 33596497

[52] McArthur LH Riggs A Uribe F Spaulding TJ. Health belief model offers opportunities for designing weight management interventions for college students. J Nutr Educ Behav. (2018) 50:485–93. doi: 10.1016/j.jneb.2017.09.010, PMID: 29097024

[53] Keshani P Hossein Kaveh M Faghih S Salehi M. Improving diet quality among adolescents, using health belief model in a collaborative learning context: a randomized field trial study. Health Educ Res. (2019) 34:279–88. doi: 10.1093/her/cyz009, PMID: 30915431

[54] Mercadante AR Law AV. Will they, or Won’t they? Examining patients’ vaccine intention for flu and COVID-19 using the health belief model. Res Soc Adm Pharm RSAP. (2021) 17:1596–605. doi: 10.1016/j.sapharm.2020.12.012, PMID: 33431259 PMC7833824

[55] Barkhordari-Sharifabad M Vaziri-Yazdi S Barkhordari-Sharifabad M. The effect of teaching puberty health concepts on the basis of a health belief model for improving perceived body image of female adolescents: a quasi-experimental study. BMC Public Health. (2020) 20:370. doi: 10.1186/s12889-020-08482-2, PMID: 32197594 PMC7083033

[56] Layman Ba HM Keirns Ms NG Hawkins PhD MAW. Internalization of body image as a potential mediator of the relationship between body acceptance by others and intuitive eating. J Am Coll Health J ACH. (2023) 71:1797–803. doi: 10.1080/07448481.2021.1947832, PMID: 34292849

[57] Linardon J Tylka TL Fuller-Tyszkiewicz M. Intuitive eating and its psychological correlates: a meta-analysis. Int J Eat Disord. (2021) 54:1073–98. doi: 10.1002/eat.23509, PMID: 33786858

[58] Falbe J Thompson HR Patel A Madsen KA. Potentially addictive properties of sugar-sweetened beverages among adolescents. Appetite. (2019) 133:130–7. doi: 10.1016/j.appet.2018.10.032, PMID: 30385262 PMC6488513

[59] Zhou J West TN Lee S-H Choi I Hitokoto H Otake K. Do people from different cultures vary in how much positive emotions resonate in day-to-day social interactions? Examining the role of relational mobility. J Cross-Cult Psychol. (2024) 55:347–67. doi: 10.1177/00220221241235926

[60] Schmader T Block K. Engendering identity: toward a clearer conceptualization of gender as a social identity. Sex Roles. (2015) 73:474–80. doi: 10.1007/s11199-015-0536-3

[61] Araiza AM Wellman JD. Weight stigma predicts inhibitory control and food selection in response to the salience of weight discrimination. Appetite. (2017) 114:382–90. doi: 10.1016/j.appet.2017.04.009, PMID: 28416329 PMC5533089

[62] Wattelez G Frayon S Cavaloc Y Cherrier S Lerrant Y Galy O. Sugar-sweetened beverage consumption and associated factors in school-going adolescents of New Caledonia. Nutrients. (2019) 11:452. doi: 10.3390/nu11020452, PMID: 30795633 PMC6412716

[63] Saghafi-Asl M Aliasgharzadeh S Asghari-Jafarabadi M. Factors influencing weight management behavior among college students: an application of the health belief model. PLoS One. (2020) 15:e0228058. doi: 10.1371/journal.pone.0228058, PMID: 32032376 PMC7006943

[64] Withers LA Vernon LL. To err is human: embarrassment, attachment, and communication apprehension. Personal Individ Differ. (2006) 40:99–110. doi: 10.1016/j.paid.2005.06.018

[65] Lastovicka JL Fernandez KV. Three paths to disposition: the movement of meaningful possessions to strangers. J Consum Res. (2005) 31:813–23. doi: 10.1086/426616

